# Supplementary material for: Pseudomonas aeruginosa Nonphosphorylated AlgR Induces Ribonucleotide Reductase Expression under Oxidative Stress Infectious Conditions
Source: mSystems. 2023 Feb 16;8(2):e01005-22. doi: 10.1128/msystems.01005-22 (PMC10134789; doi:10.1128/msystems.01005-22)

Fig. S1. Multiple alignment of the class II and I RNR promoter regions. Alignment of the promoter regions of A) *nrdJ* and B) *nrdA* from the *P. aeruginosa* PAO1, PA14, and PAET1 strains. The AlgR binding box sites found in the promoter regions of the RNR are indicated with black rectangles.

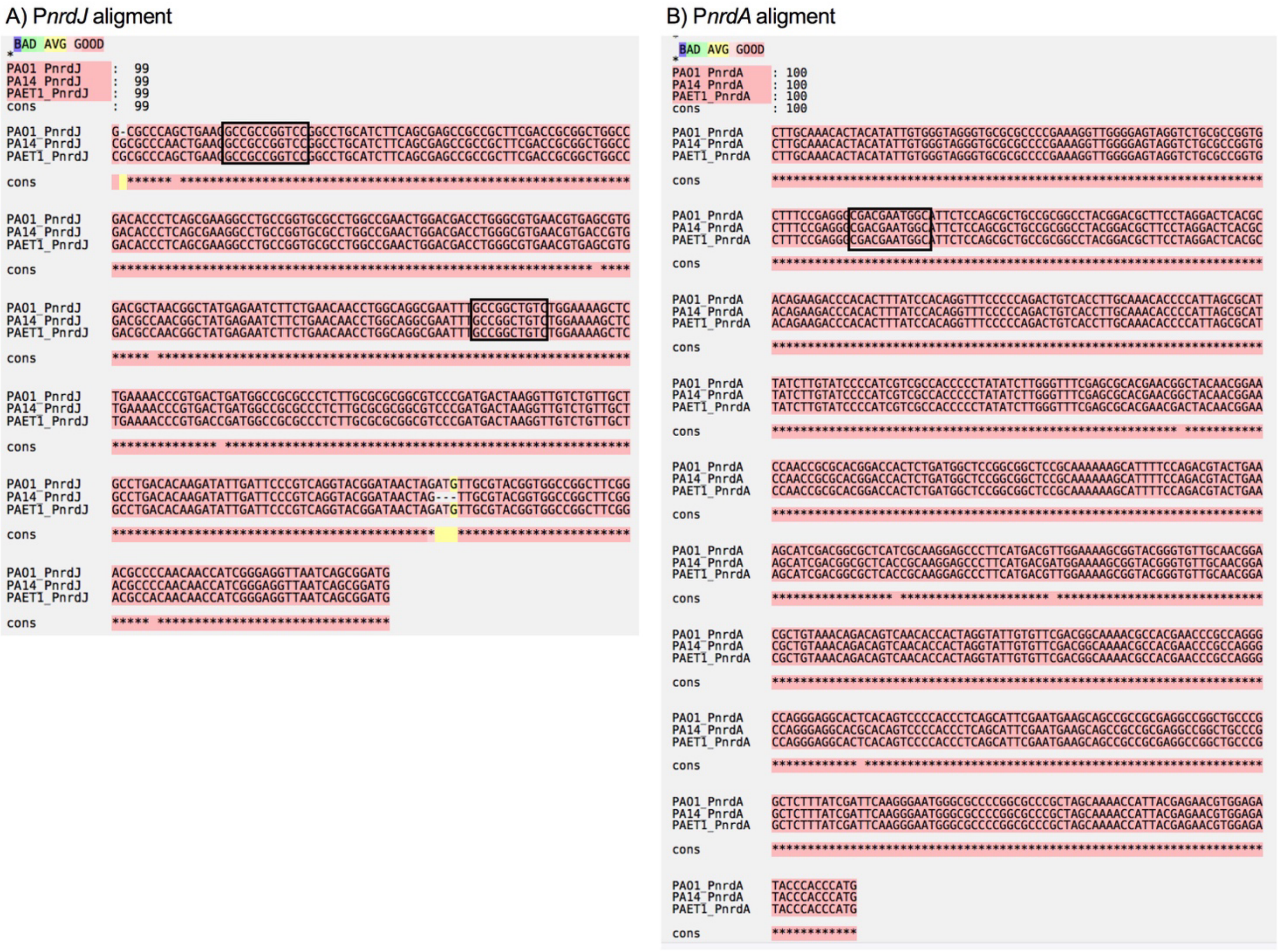

Supplement: FIG S1 [file msystems.01005-22-s0001.pdf]
