# Supplementary material for: Pseudomonas aeruginosa Nonphosphorylated AlgR Induces Ribonucleotide Reductase Expression under Oxidative Stress Infectious Conditions
Source: mSystems. 2023 Feb 16;8(2):e01005-22. doi: 10.1128/msystems.01005-22 (PMC10134789; doi:10.1128/msystems.01005-22)
Supplement: TABLE S1 [file msystems.01005-22-s0002.pdf]

Table S1. Bacterial strains and plasmids used in this study

Name	Reference name	Description	Source
**Plasmids**			
pJET1.2/blunt	pJET1.2b	Blunt-end vector; AmpR	Thermo Scientific
pETS130-GFP	pETS130	Broad host range, promoterless GFP; GmR	(1)
pETS134	pETS-P*A*	pETS130 derivative carrying *nrdA* promoter; GmR	(1)
pETS208	pETS-P*A*-Δbox1	pETS130 derivative carrying AlgR-box1 mutation in P*nrdA*, GmR	(2)
pETS180	pETS-P*J*	pETS130 derivative carrying *nrdJ* promoter; GmR	(3)
pETS211	pETS-P*J*-Δbox1+2	pETS130 derivative carrying AlgR-box1+2 mutation in P*nrdJ*, GmR	(2)
pET229	pETS-P*katA*	pETS130 derivative carrying *katA* promoter; GmR	This work
pETS230	pETS-P*katB*	pETS130 derivative carrying *katB* promoter; GmR	This work
pETS203	pUCP-AlgR	pUCP20T derivative carrying the *algR* gene; CbR	(2)
pETS204	pUCP-D54N	pUCP20T derivative carrying the *algR*D54N gene; CbR	(2)

pETS207	pETS-P*fimS*	pETS130 derivative carrying *fimS-algR* promoter; GmR	(2)
pETS231	pETS-P*algR*	pETS130 derivative carrying *algR* promoter; GmR	This work
pETS232	pETS-P*J*-ΔAnrbox	pETS130 derivative carrying Anr-box mutation in P*nrdJ*, GmR	This work
pETS233	pETS-P*J*-Δbox1+2-ΔAnrbox	pETS130 derivative carrying AlgR-box1+2 and Anr-box mutation in P*nrdJ*, GmR	This work
pETS220	pLUX	Broad host range, promoterless luxCDABE; GmR	(4)
pETS221	pLUX-P*A*	pETS220 derivative carrying *nrdA* promoter; GmR	(4)
pETS234	pLUX-P*A*-Δbox1	pETS220 derivative carrying AlgR-box1 mutation in P*nrdA*, GmR	This work
pETS222	pLUX-P*J*	pETS220 derivative carrying *nrdJ* promoter; GmR	(4)
pETS235	pLUX-P*J*-Δbox1+2	pETS220 derivative carrying AlgR-box1+2 mutation in P*nrdJ*, GmR	This work
pETS236	pLUX-P*J*-ΔAnrbox	pETS220 derivative carrying AnrR-box mutation in P*nrdJ*, GmR	This work
pETS237	pLUX-P*J*-Δbox1+2-ΔAnrbox	pETS220 derivative carrying AlgR-box1+2 and Anr-box mutation in P*nrdJ*, GmR	This work
pETS238	pLUX-P*katA*	pETS220 derivative carrying *katA* promoter; GmR	This work
pETS239	pLUX-P*katB*	pETS220 derivative carrying *katB* promoter; GmR	This work
pETS240	pLUX-P*algR*-1	pETS220 derivative carrying *fimS-algR* promoter; GmR	This work
pETS241	pLUX-P*algR*-2	pETS220 derivative carrying *algR* promoter; GmR	This work

pETS225	pLUX-*anr*	pETS220 derivative carrying *anr* gene fragment; GmR	(4)
pEX100Tlink	pEX100Tlink	pEX100T with a MCS; AmpR	(5)
pETS242	pEX100Tlink::*algR*′-`*algR* (PA14)	pEX100Tlink containing 5′ and 3′ flanking sequences of *algR* of *P. aeruginosa* PA14; AmpR	This work
pETS243	pEX100Tlink::*algR*′-`*algR* (PAET1)	pEX100Tlink containing 5′ and 3′ flanking sequences of *algR* of *P. aeruginosa* PAET1; AmpR	This work
pETS244	pEX100Tlink::*algR*′-Gm*lox*-`*algR* (PA14)	pEX100Tlink containing 5′ and 3′ flanking sequences of *algR* :: Gm*lox* of *P. aeruginosa* PA14; AmpR, GmR	This work
pETS245	pEX100Tlink::*algR*′-Gm*lox*-`*algR* (PAET1)	pEX100Tlink containing 5′ and 3′ flanking sequences of *algR* :: Gm*lox* of *P. aeruginosa* PAET1; AmpR, GmR	This work
pUCGmlox	pUCGmlox	pUC18-based vector containing the *lox* flanked *aacC1* gene	(5)
pCM157	pCM157	*cre* expression vector; TcR	(6)
**Strains**			
E. coli			

DH5α	DH5α	*recA1 endA1 hsdR17 supE44 thi-1 relA1 Δ(lacZYA-argF)U169 deoR Φ80dlacZM15*	Laboratory
S17.1	S17.1	*recA thi pro hsdR- M+RP4::2-Tc::Mu::Km Tn7 Tpr Smr Xpir*	(7)
P. aeruginosa			
PAO1	PAO1 WT	Wild-type (ATCC 15692 / CECT 4122) - Spanish Type Culture Collection	Laboratory
PW9855	PAO1 Δ*algR*	*P. aeruginosa* PAO1 algR::ISphoA/hah; TcR	(8)
PA14	PA14 WT	*P. aeruginosa* PA14 wild-type strain	Laboratory
pETS131	PA14 Δ*algR*Gm*lox*	*P. aeruginosa* PA14Δ*algR*::Gm*lox*	This work
pETS132	PA14 Δ*algR*	*P. aeruginosa* PA14Δ*algR*::*lox*	This work
PAET1	PAET1 WT	*P. aeruginosa* clinical isolated from CF patient strain	(9)
pETS133	PAET1 Δ*algR*Gm*lox*	*P. aeruginosa* PAET1Δ*algR*::Gm*lox*	This work
pETS134	PAET1 Δ*algR*	*P. aeruginosa* PAET1Δ*algR*::*lox*	This work

1. Sjöberg BM, Torrents E. 2011. Shift in ribonucleotide reductase gene expression in *Pseudomonas aeruginosa* during infection. Infect Immun 79:2663-9.
2. Crespo A, Pedraz L, Van Der Hofstadt M, Gomila G, Torrents E. 2017. Regulation of ribonucleotide synthesis by the *Pseudomonas aeruginosa* two-component system AlgR in response to oxidative stress. Sci Rep 7:17892.

3. Crespo A, Pedraz L, Torrents E. 2015. Function of the *Pseudomonas aeruginosa* NrdR Transcription Factor: Global Transcriptomic Analysis and Its Role on Ribonucleotide Reductase Gene Expression. PLoS One 10:e0123571.

4. Moya-Anderico L, Admella J, Fernandes R, Torrents E. 2020. Monitoring Gene Expression during a *Galleria mellonella* Bacterial Infection. Microorganisms 8.

5. Quenee L, Lamotte D, Polack B. 2005. Combined *sacB*-based negative selection and cre-lox antibiotic marker recycling for efficient gene deletion in *Pseudomonas aeruginosa*. Biotechniques 38:63-7.

6. Marx CJ, Lidstrom ME. 2002. Broad-host-range *cre-lox* system for antibiotic marker recycling in Gram-negative bacteria. Biotechniques 33:1062-7.

7. de Lorenzo V, Cases I, Herrero M, Timmis KN. 1993. Early and late responses of TOL promoters to pathway inducers: identification of postexponential promoters in *Pseudomonas putida* with *lacZ-tet* bicistronic reporters. J Bacteriol 175:6902-7.

8. Jacobs MA, Alwood A, Thaipisuttikul I, Spencer D, Haugen E, Ernst S, Will O, Kaul R, Raymond C, Levy R, Chun-Rong L, Guenthner D, Bovee D, Olson MV, Manoil C. 2003. Comprehensive transposon mutant library of *Pseudomonas aeruginosa*. Proc Natl Acad Sci U S A 100:14339-44.

9. Crespo A, Gavalda J, Julian E, Torrents E. 2017. A single point mutation in class III ribonucleotide reductase promoter renders *Pseudomonas aeruginosa* PAO1 inefficient for anaerobic growth and infection. Sci Rep 7:13350.
