# Supplementary material for: Pseudomonas aeruginosa Nonphosphorylated AlgR Induces Ribonucleotide Reductase Expression under Oxidative Stress Infectious Conditions
Source: mSystems. 2023 Feb 16;8(2):e01005-22. doi: 10.1128/msystems.01005-22 (PMC10134789; doi:10.1128/msystems.01005-22)
Supplement: TABLE S2 [file msystems.01005-22-s0003.pdf]

Table S2. Primers and probes used in this study.

Primers

Number	Name	Sequence (5' -> 3')	Application
1	PkatA_BamHI_Fw	GGATCCGAATTGAACCATGCGCCATC	P*katA* promoter cloning/Sequencing
2	PkatA_SmaI_Rv	CCCGGGTCTGCACGTTCTGGTTA	P*katA* promoter cloning/Sequencing
3	PkatB_BamHI_Fw	GGATCCCAGATGCCGGAAGTTGAAGT	P*katB* promoter cloning/Sequencing
4	PkatB_SmaI_Rv	CCCGGGTCGAAACGTTGCAACTTC	P*katB* promoter cloning/Sequencing
5	fimS_SacI_Fw	GAGCTCCTGTTCTGCCGCCTG	PalgR promoter cloning/Sequencing
6	fimS_BamHI_Rv	GGATCCCATCGACAATCAGGACATTCA	PalgR promoter cloning/Sequencing
7	PnrdJ_PAO_BamHI_up_new	GGATCCCGCGCCCAGCTGAAGG	Mutagenesis P*nrdJ* Anrbox/Sequencing
8	PJ mut ad low	TATCCGTACCTGCGTGGAATCAATA	Mutagenesis P*nrdJ* Anrbox
9	PJ mut ad up	TATTGAGGACACGCAGGTACGGA	Mutagenesis P*nrdJ* Anrbox
10	PnrdJ_SmaI_Rv	CCCGGGGACTGCGTGCGTCTGTC	Mutagenesis P*nrdJ* Anrbox/Sequencing
11	fimS_HindIII_Fw	AAGCTTGCAGGTCGAGGCGGTTTAT	Deletion *algR* gene

12	fimS_BamHI_Rv	GGATCCCATCGACAATCAGGACATTCA	Deletion *algR* gene
13	HemC_BamHI_Fw	GGATCCGCATTTCCAGCTCTACCT	Deletion *algR* gene
14	HemC_SacI_Rv	GAGCTCGATGGTGTAGAGGCCGA	Deletion *algR* gene
15	pJET 1.2 fw	CGACTCACTATAGGGAGAGCGGC	Check cloning/Sequencing
16	pJET 1.2 rv	AAGAACATCGATTTTCCATGGCAG	Check cloning/Sequencing
17	pUCP20T-up	CCTCTTCGCTATTACGCCAG	Check cloning/Sequencing
